# Neuroanatomical restoration of salience network links reduced headache impact to cognitive function improvement in mild traumatic brain injury with posttraumatic headache

**DOI:** 10.1186/s10194-023-01579-0

**Published:** 2023-04-21

**Authors:** Hui Xu, Cheng Xu, Pengpeng Gu, Yike Hu, Yunyu Guo, Guanghui Bai

**Affiliations:** 1 Department of Radiology, The Second Affiliated Hospital and Yuying Children’s Hospital of Wenzhou Medical University, Wenzhou, 325027 Zhejiang China; 2 Peter Boris Centre for Addictions Research, St. Joseph’s Healthcare Hamilton/McMaster University, 100 West 5Th Street, Hamilton, ON L8P 3R2 Canada; 3 School of Psychology and Cognitive Science, East China Normal University, Shanghai, 200062 China; 4 Department of Physical Medicine and Rehabilitation, The Second Affiliated Hospital and Yuying Children’s Hospital of Wenzhou Medical University, Wenzhou, 325027 Zhejiang China

**Keywords:** Neuroanatomical restoration, Salience network, Mild traumatic brain injury, Headache impact, Cognitive function

## Abstract

**Background:**

Neuroanatomical alterations have been associated with cognitive deficits in mild traumatic brain injury (MTBI). However, most studies have focused on the abnormal gray matter volume in widespread brain regions using a cross-sectional design in MTBI. This study investigated the neuroanatomical restoration of key regions in salience network and the outcomes in MTBI.

**Methods:**

Thirty-six MTBI patients with posttraumatic headache (PTH) and 34 matched healthy controls were enrolled in this study. All participants underwent magnetic resonance imaging scans and were assessed with clinical measures during the acute and subacute phases. Surface-based morphometry was conducted to get cortical thickness (CT) and cortical surface area (CSA) of neuroanatomical regions which were defined by the Desikan atlas. Then mixed analysis of variance models were performed to examine CT and CSA restoration in patients from acute to subacute phase related to controls. Finally, mediation effects models were built to explore the relationships between neuroanatomical restoration and symptomatic improvement in patients.

**Results:**

MTBI patients with PTH showed reduced headache impact and improved cognitive function from the acute to subacute phase. Moreover, patients experienced restoration of CT of the left caudal anterior cingulate cortex (ACC) and left insula and cortical surface area of the right superior frontal gyrus from acute to subacute phase. Further mediation analysis found that CT restoration of the ACC and insula mediated the relationship between reduced headache impact and improved cognitive function in patients.

**Conclusions:**

These results showed that neuroanatomical restoration of key regions in salience network correlated reduced headache impact with cognitive function improvement in MTBI with PTH, which further substantiated the vital role of salience network and provided an alternative clinical target for cognitive improvement in MTBI patients with PTH.

**Graphical Abstract:**

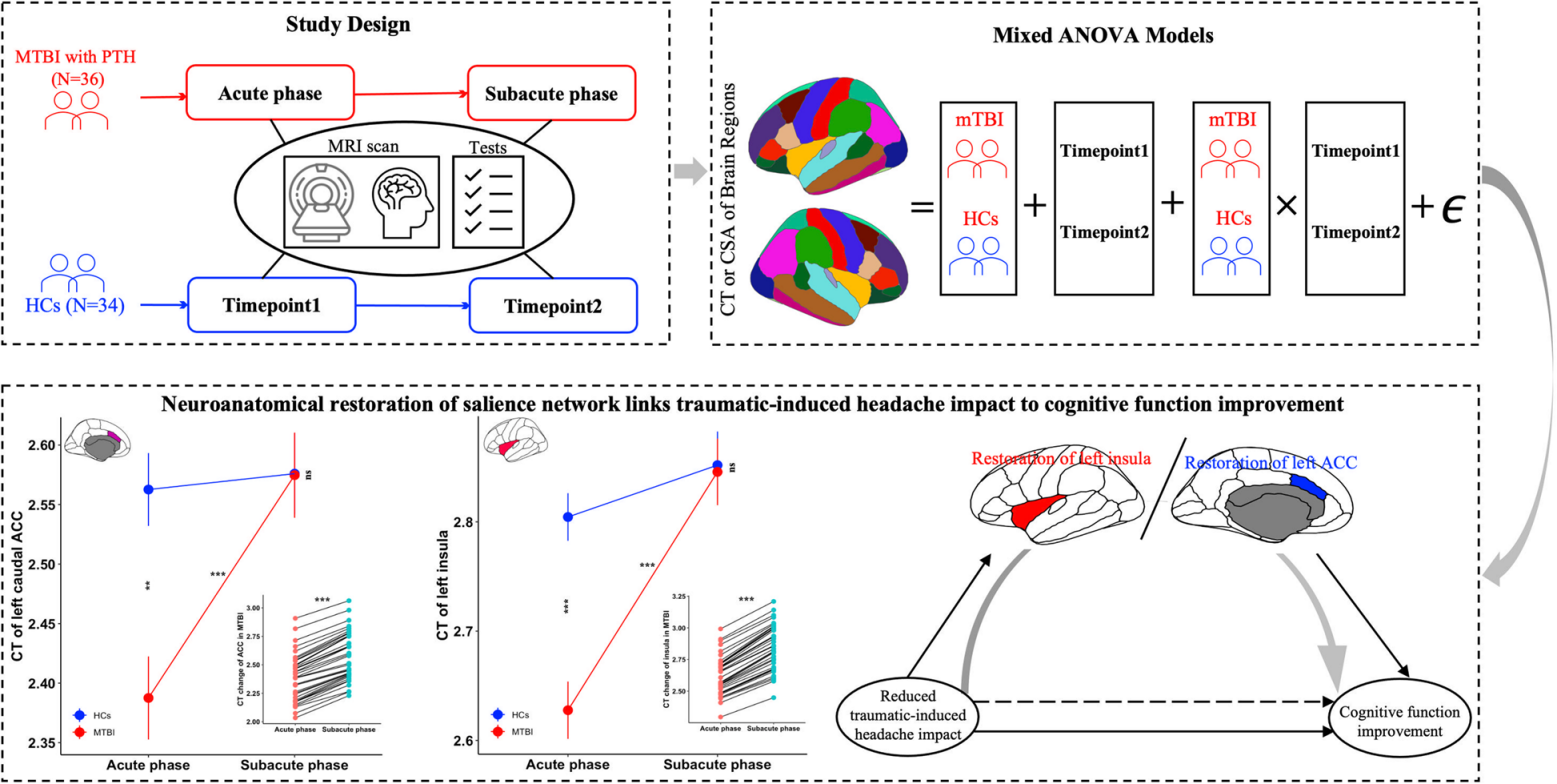

**Supplementary Information:**

The online version contains supplementary material available at 10.1186/s10194-023-01579-0.

## Background

Traumatic brain injury (TBI) refers to any damage or alteration of normal brain function caused by an external mechanical force [1], which causes temporary or permanent impairment of physical, mental, cognitive and emotional function [2]. An estimated 69 million people worldwide suffer TBI each year [3], and mild TBI (MTBI) accounts for approximately 80–90% of all TBI cases [4, 5], although the actual incidence is likely to be higher as many patients with MTBI are not treated in hospital [6]. As a high prevalence of trauma or pain related symptom, posttraumatic headache (PTH) was likely to developed into chronic pain in MTBI [7]. Clinical symptoms of MTBI include short periods of unconsciousness, headache, dizziness, irritability, anxiety, impaired concentration, and cognitive function deficits [8, 9]. Although most patients with MTBI can recover fully, many studies have found that MTBI can induce long-term physical and cognitive dysfunction [10, 11], including reduced information processing speed, chronic pain [12], and depression [13], which can have lasting negative effects not only on individuals but also on families and society.

The past few years have witnessed a burgeoning interest in implementing magnetic resonance imaging (MRI) techniques to assess the relationship between clinical symptoms of MTBI and abnormal brain morphometry. Previous studies found that MTBI patients showed lower grey matter volume (GMV) in widespread brain regions, including the frontal, precuneus, and temporal lobes [14, 15]. It has also been suggested that MTBI was associated with significantly lower GMV in the right insula, contributing to poor attention [16]. Another long-term follow-up study found that MTBI was associated with less GMV of parietal lobes and a more severe persistent post-concussion syndrome [17]. Moreover, another study found that MTBI patients showed increased GMV of the ventral medial prefrontal cortex and right cingulate gyrus, with performance improvement in visuospatial design fluency and emotional functioning [18]. However, these studies mainly focused on GMV (a neuroanatomical measure) and its correlations with clinical symptoms in MTBI. Previous research has demonstrated that GMV can be separated into two distinct morphological features of cortical architecture: cortical thickness (CT) and cortical surface area (CSA) [19]. Given that CT and CSA have distinct developmental trajectories and uncorrelated genetic backgrounds [20], they should be considered separate morphometric features of neurodevelopment [21, 22].

However, few studies on surface-based morphometry found that MTBI patients had lower CT in the right temporal lobe and left insula and lower CSA in frontal regions [23, 24]. Additionally, previous research has found a significant reduction in frontal CT in MTBI patients post 3 months-injury, suggesting a potential link to the post-traumatic inflammatory response and local micro edema [25]. In contrast, another study found no difference in CT or CSA of brain regions between patients and healthy subjects in the early post-trauma phase of brain trauma [26]. However, given the cross-sectional design of these studies, the causal relationship or temporal sequence of the observed neuroanatomical changes could not be determined.

To address these limitations in the literature, the current study conducted surface-based morphometry and mixed analysis of variance (ANOVA) models to investigate neuroanatomical restoration in MTBI with PTH from the acute to subacute phases. Furthermore, this study established mediation effects models to explore the relationships between neuroanatomical restoration and symptomatic improvement. Importantly, we assessed whether neuroanatomical restoration of certain regions could be observed in MTBI from the acute to subacute phase and identified the effect of neuroanatomical restoration on symptomatic improvement.

## Methods

### Participants

A total of seventy right-handed participants enrolled in this study, including 36 MTBI patients with PTH and 34 matched healthy controls (HCs). To determine the required sample sizes for sufficient power for the mixed ANOVA model, a priori power analysis was conducted using G*Power software (version 3.1) with a significant level at 0.05 by setting the statistical power at 0.95, which yielded a minimum sample size of 33 for each group. Patients were recruited from the local emergency department of the local hospital. The diagnosis of MTBI was established by two experienced neurologists in accordance with the guidelines of the World Health Organization’s Collaborating Centre for Neurotrauma Task [27–29]. To be included in this study, patients had to have fulfilled the following: (1) with Glasgow Coma Scale score of 13–15; (2) one or more of the following: loss of consciousness (if present) < 30 min, post-traumatic amnesia (if present) < 24 h, and/or other transient neurological abnormalities such as focal signs, seizure, and intracranial lesion not necessitating surgery. (3) PTH was assessed according to the Third Edition of the International Classification of Headache Disorders [30]. Those with a history of neurological diseases, psychiatric condition, head injury, substance or alcohol abuse, intubation, and/or presence of a skull fracture and were administered sedatives on arrival in the emergency department, spinal cord injury, MTBI from other injuries (e.g., systemic injuries, facial injuries, or intubation), or other problems (e.g., psychological trauma, language barrier, or coexisting medical conditions), or from craniocerebral injury, were all excluded from the study. All MTBI patients have only received same amounts of Vitamin B_12_ to enhance nerve repair, which has been suggested useful as a novel neuroprotective drug for TBI in mice models [31].

**Fig. 1:**
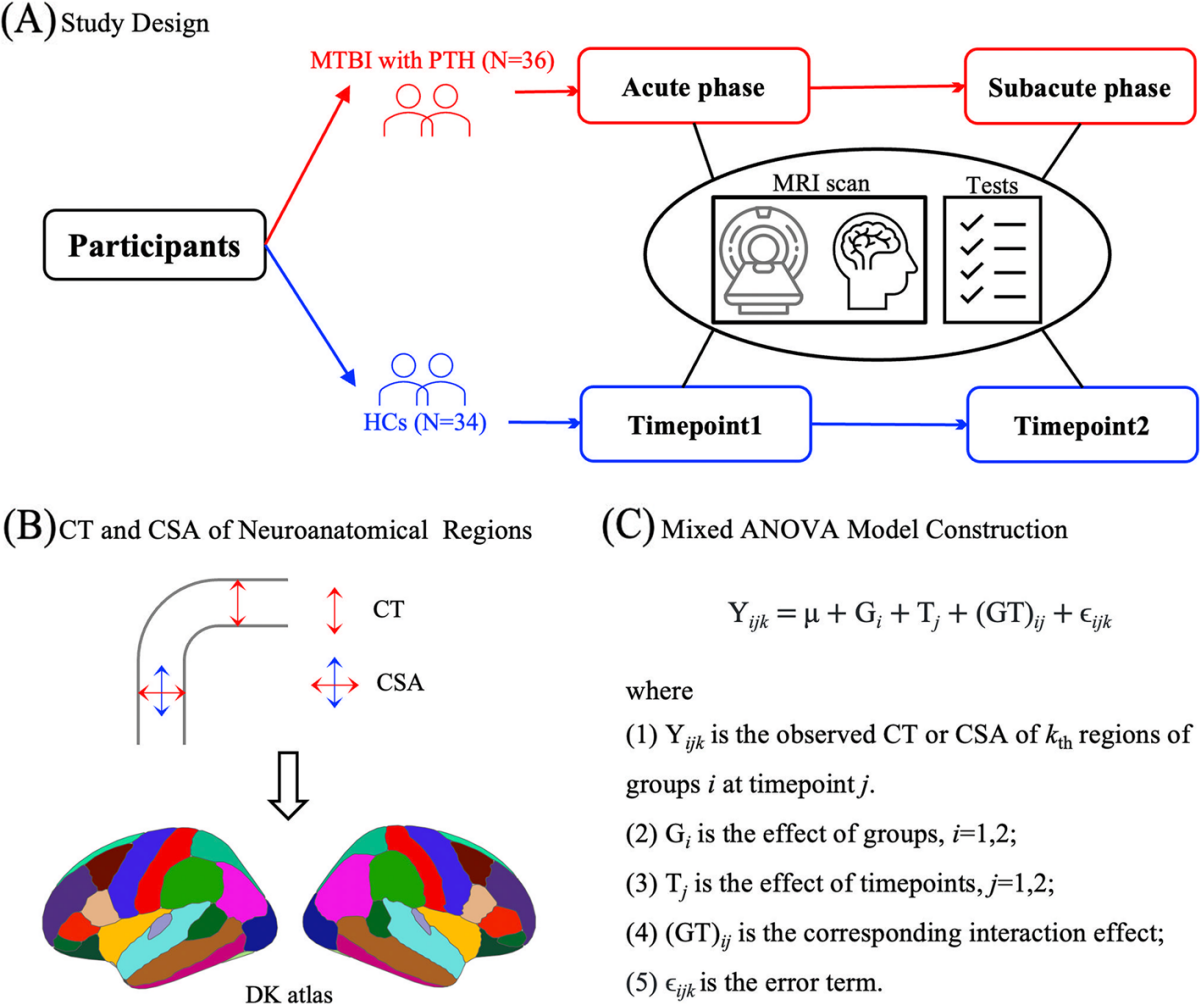
Study design framework. **A** Participants recruited in this study. The MRI scanning and neuropsychological tests were performed at two timepoints for patients and matched HCs. **B** Construction of CT and CSA of cortical regions and defined by the Desikan atlas. **C** Mixed ANOVA model was conducted for CT and CSA of 68 bilateral cortical regions. MTBI, mild traumatic brain injury; PTH, posttraumatic headache; HCs, healthy controls; CT, cortical thickness; CSA, cortical surface area; ANOVA, analysis of variance

MRI scanning for patients was conducted within seven days post-injury (acute phase) and one-month post-injury (subacute phase). Neuropsychological tests and clinical symptoms assessments were performed within 48 h of MRI scanning. The HCs underwent MRI scanning and neuropsychological tests at the same time points (Fig. 1A). All participants provided informed consent, and this study was approved by the local ethics committee in accordance with the Declaration of Helsinki. The study design framework of this study is outlined in Fig. 1.

### Neuropsychological and clinical symptoms assessments

To examine cognitive function, neuropsychological tests were conducted in this study, including (1) Trail-Making Test Part-A (TMT-A) for rote memory assessment; (2) Forward Digit Span (FDS) and Backward Digit Span (BDS) test of Wechsler Adult Intelligence Scale-III for working memory assessment; (3) Digit Symbol Coding (DSC) task for the assessments of cognitive function and information processing speed. Additionally, self-reported clinical symptom assessments were performed, including (1) the Insomnia Severity Index (ISI) for sleep quality; (2) the short-form Headache Impact Test (HIT) for a measure of trauma-induced headache impact in patients. All neuropsychological and clinical symptom assessments were performed by an experienced clinical psychologist blinded to the clinical data of patients.

### MRI data acquisition

The MRI scanning for participants was conducted using a 3.0 T MRI scanner (GE 750 Medical Systems). During the scanning, a custom-built head holder was used to hold the participants’ heads in position. Standard T1-weighted 3D anatomical data were acquired using a three-dimensional magnetization-prepared rapid gradient echo sequence (echo time $=$ 3.17 ms, repetition time $=$ 8.15 ms, flip angle $=$ 9°, slice thickness $=$ 1 mm, field of view $=$ 256 mm $\times$ 256 mm, matrix size $=$ 256 $\times$ 256, acquisition time $=$ 4 min, 30 s). All participants were asked to relax with closed eyes and not engage in cognitive or motor-related activity. The alertness of the participants during the scan was confirmed immediately afterward.

Additional neuroimaging data (including T1-flair, T2- flair, T2, and susceptibility-weighted imaging) were captured and used to identify focal lesions and cerebral microbleeds. No contusions were detected in any patients.

### MRI data preprocessing

Structural T1-weighted MRI data of every participant was processed with FreeSurfer v7.2.0 software package (http://surfer.nmr.mgh.harvard.edu). The details of surface-based morphology analysis were documented in previous studies [32–34]. Briefly, the FreeSurfer pipeline processing included motion correction, removal of non-brain tissue, automated Talairach transformation of each participant’s native brain, intensity normalization, tessellation of the gray/white matter boundary, automated topology correction, surface deformation following intensity gradients, registration of the participant’s native brain to a common spherical atlas, and cortical surface reconstruction. The cortical morphologies were smoothed using a 10 mm full-width-at-half-maximum Gaussian kernel to obtain CT and CSA measurements based on previous research [35–37]. The cortical morphologies were smoothed using a 10 mm full-width-at-half-maximum Gaussian kernel to obtain CT and CSA measurements. CT was calculated at each vertex in the cortex by measuring the distance between the pial and gray-white matter surfaces. CSA was estimated by averaging the area of all faces connected to a given vertex on the white matter surface. During preprocessing, all outputs were inspected for accuracy, and manual corrections were made where needed. Then, the average value of CT and CSA within 34 automatically cortical parcellations was defined by the Desikan atlas [38] in each hemisphere (Fig. 1B). Finally, the values of these cortical surface indices for each cortical region were exported for analysis.

### Statistical analyses

#### Statistical analyses of demographic and clinical data

For demographic data, independent two samples t-test was conducted to evaluate group differences in age and education level, whereas a chi-square test was used to assess sex difference between groups. To analyze the neuropsychological and clinical symptom data, we used a mixed ANOVA model with two factors: groups (patients and HCs) as the between-subjects factor and timepoints (acute and subacute phases) as the within-subjects factor. The test scores were used as the dependent variable in the analysis. Then, a simple effect analysis was performed with Bonferroni corrections. A *P*-value < 0.05 was statistically significant.

#### Statistical analyses of MRI data

For both CT and CSA, a mixed ANOVA model was used to assess the interactions between different groups (patients, HCs) and time points (acute phase, subacute phase) in the 68 bilateral cortical regions (Fig. 1C). Further, to estimate the proportion of variance associated with each main effect and interaction effect in the mixed model, eta squared (η^2^) was calculated as a measure of the effect size [39]. Then, a simple effect analysis was performed with Bonferroni corrections. A *P*-value < 0.05 was statistically significant.

#### Correlation analyses and mediation analyses

The simple effect analyses found that patients showed cognitive function improvement (indexed by DSC), reduced headache impact (indexed by HIT), and CT or CSA restoration of several key regions. To investigate their relationships, correlation analyses were performed between their change in patients from acute to subacute phase after stratifying for age, sex, and education level as covariates. The significance threshold was set at *P* < 0.05.

Before the correlation analyses, the percentage of change in patients was calculated using the following formula:

$${\mathrm{Variable}}_{\mathrm{change }}= [({\mathrm{Variable}}_{\mathrm{subacute}-\mathrm{phase}} - {\mathrm{Variable}}_{\mathrm{acute}-\mathrm{phase}})/{\mathrm{Variable}}_{\mathrm{acute}-\mathrm{phase}}] \times 100\mathrm{\%}$$

where the variable was DSC, HIT, CT of the left caudal anterior cingulate cortex (ACC) and left insula, and CSA of the right superior frontal gyrus (SFG) based on the simple effect analyses. Hence, reduced headache impact was indexed by the percent change of the score of HIT in patients from acuate to subacute phases; cognitive function improvement was indexed by the percent change of the score of DSC in patients from acuate to subacute phases; neuroanatomical restoration of ACC, insula, and SFG were indexed by the percent change of the CT of ACC, CT of insula, and CSA of SFG in patients from acuate to subacute phases respectively.

Further, mediation effects models were built based on significant results of the above correlation analyses to explore whether the restoration of neuroanatomical regions influenced the relationship between the reduced headache impact and cognitive function improvement in patients. The bias-corrected 95% confidence interval (CI) was calculated to estimate the significance of indirect and direct effects using the bootstrapping procedure based on 10,000 bootstrap samples [40]. The mediation was statistically significant when the bootstrapped 95% CI did not include zero [41]. Three mediation models were established using “reduced headache impact” as the independent variable and “cognitive function improvement” as the outcome. The restoration of the left insula, left ACC, and right SFG were examined as mediators, respectively, using a regression-based approach.

## Results

### Demographic and clinical characteristics

**Fig. 2:**
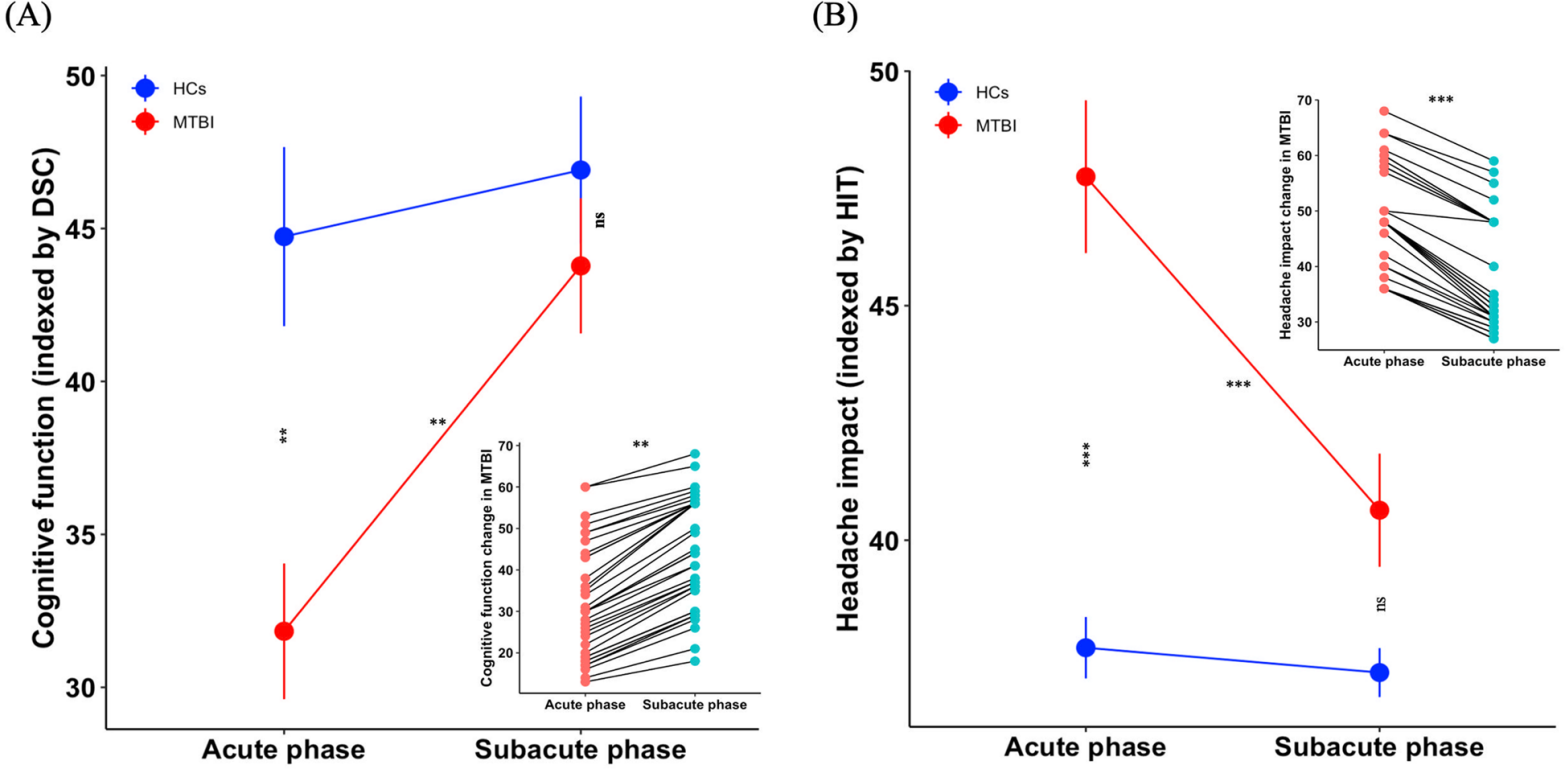
Clinical symptoms changes in MTBI patients with PTH from acute to subacute phase related to HCs. **A** Cognitive function (indexed by score of DSC) improvement in patients from acute to subacute phase. The subpanel shows cognitive function change for each patient from acute to subacute phase, which indicates significant cognitive function improvement from acute to subacute phase in patients. **B** Reduced traumatic-induced headache impact (indexed by score of HIT) in patients from acute to subacute phase. The subpanel shows headache impact change for each patient from acute to subacute phase, which indicates significant reduced headache impact from acute to subacute phase in patients. MTBI, mild traumatic brain injury; PTH, posttraumatic headache; HCs, healthy controls; DSC, Digit Symbol Coding; HIT, the short-form Headache Impact Test. ^**^, *P* < 0.01; ^***^, *P* < 0.001; ns, not significant. Data are presented as mean ± SEM

Demographic and clinical data for patients and HCs are summarized in Table 1. No significant differences in age, sex, and years of education were observed between groups (all *P* $>$ 0.05). In terms of cognitive function, there was a significant interaction effect found for DSC (*F*_(1, 68)_ $=$ 4.36, *P *$<$ 0.05), and simple effect analysis found that during the acute phase, patients exhibited significantly poorer cognitive function related to HCs (*P* $<$ 0.01), but there was no significant difference between groups during the subacute phase (Fig. 2A), which indicated cognitive function improvement from the acute to the subacute phase. In terms of clinical symptoms, there was a significant interaction effect for HIT (*F*_(1, 68)_ = 12.91, *P* $<$ 0.01), and simple effect analysis found that patients suffered more serious trauma-induced headache impact than HCs (*P* $<$ 0.001) during the acute phase, but no significant difference was found between groups during the subacute phase (Fig. 2B), which implied that trauma-induced headache was reduced from the acute to subacute phase.

**Table 1:** Demographic and clinical characteristics of MTBI patients with PTH and HCs

Characteristics	Groups				Statistics					
	MTBI patients with PTH (*n* = 36)		HCs (*n* = 34)		*t/χ*^*2*^			*P-value*		
Age (years)	37.64 (2.08)		34.85 (1.87)		*t* = *0.99*			*P* = *0.33*		
Sex (F/M)	17/19		20/14		*χ*^*2*^ = *0.94*			*P* = *0.33*		
Education level (years)	10.67 (0.71)		11.68 (1.01)		*t* = *-0.82*			*P* = *0.41*		
Handedness (L/R)	0/36		0/34							
*Timepoints*	*Acute phase*	*Subacute phase*	*Acute phase*	*Subacute phase*	*F*-value			*P*-value		
*Cognitive function*										
Score of TMA-A	60.36 (5.60)	49.75 (5.92)	43.91 (5.87)	38.38 (4.11)	*F*_Groups_ = 6.52	*F*_Timepoints_ = 2.24	*F*_groups*Timepoints_ = 0.22	*P*_Groups_ = 0.01	*P*_Timepoints_ = 0.14	*P*_groups*Timepoints_ = 0.64
Score of DSC	31.83 (2.22)	43.78 (2.21)	44.74 (2.92)	46.91 (2.40)	*F*_Groups_ = 10.75	*F*_Timepoints_ = 8.68	*F*_groups*Timepoints_ = 4.36	*P*_Groups_ < 0.01	*P*_Timepoints_ = 0.003	*P*_groups*Timepoints_ < 0.05
Score of FDS	7.69 (0.25)	8.28 (0.27)	8.41 (0.25)	8.88 (0.28)	*F*_Groups_ = 6.32	*F*_Timepoints_ = 4.05	*F*_groups*Timepoints_ = 0.04	*P*_Groups_ = 0.01	*P*_Timepoints_ = 0.04	*P*_groups*Timepoints_ = 0.83
Score of BDS	3.72 (0.23)	4.33 (0.33)	4.47 (0.32)	4.50 (0.28)	*F*_Groups_ = 2.45	*F*_Timepoints_ = 1.27	*F*_groups*Timepoints_ = 0.99	*P*_Groups_ = 0.12	*P*_Timepoints_ = 0.26	*P*_groups*Timepoints_ = 0.32
*Clinical symptoms*										
Score of ISI	7.25 (1.01)	4.78 (0.91)	2.32 (0.58)	1.68 (0.45)	*F*_Groups_ = 26.17	*F*_Timepoints_ = 4.09	*F*_groups*Timepoints_ = 1.35	*P*_Groups_ < 0.001	*P*_Timepoints_ = 0.04	*P*_groups*Timepoints_ = 0.24
Score of HIT	47.75 (1.63)	40.64 (1.21)	37.71 (0.65)	37.18 (0.52)	*F*_Groups_ = 36.38	*F*_Timepoints_ = 12.23	*F*_groups*Timepoints_ = 12.91	*P*_Groups_ < 0.001	*P*_timepoints_ < 0.01	*P*_groups*Timepoints_ < 0.01

Values presented as Mean (Standard Error of the Mean) unless otherwise stated. *MTBI* mild traumatic brain injury, *PTH* posttraumatic headache, *HCs* healthy controls, *TMT-A* Trail-Making Test Part-A, *FDS* Forward Digit Span, *BDS* Backward Digit Span, *DSC* Digit Symbol Coding, *ISI* the Insomnia Severity Index, *HIT* the short-form Headache Impact Test

### Interaction effects of CT and CSA in different neuroanatomical regions

**Fig. 3:**
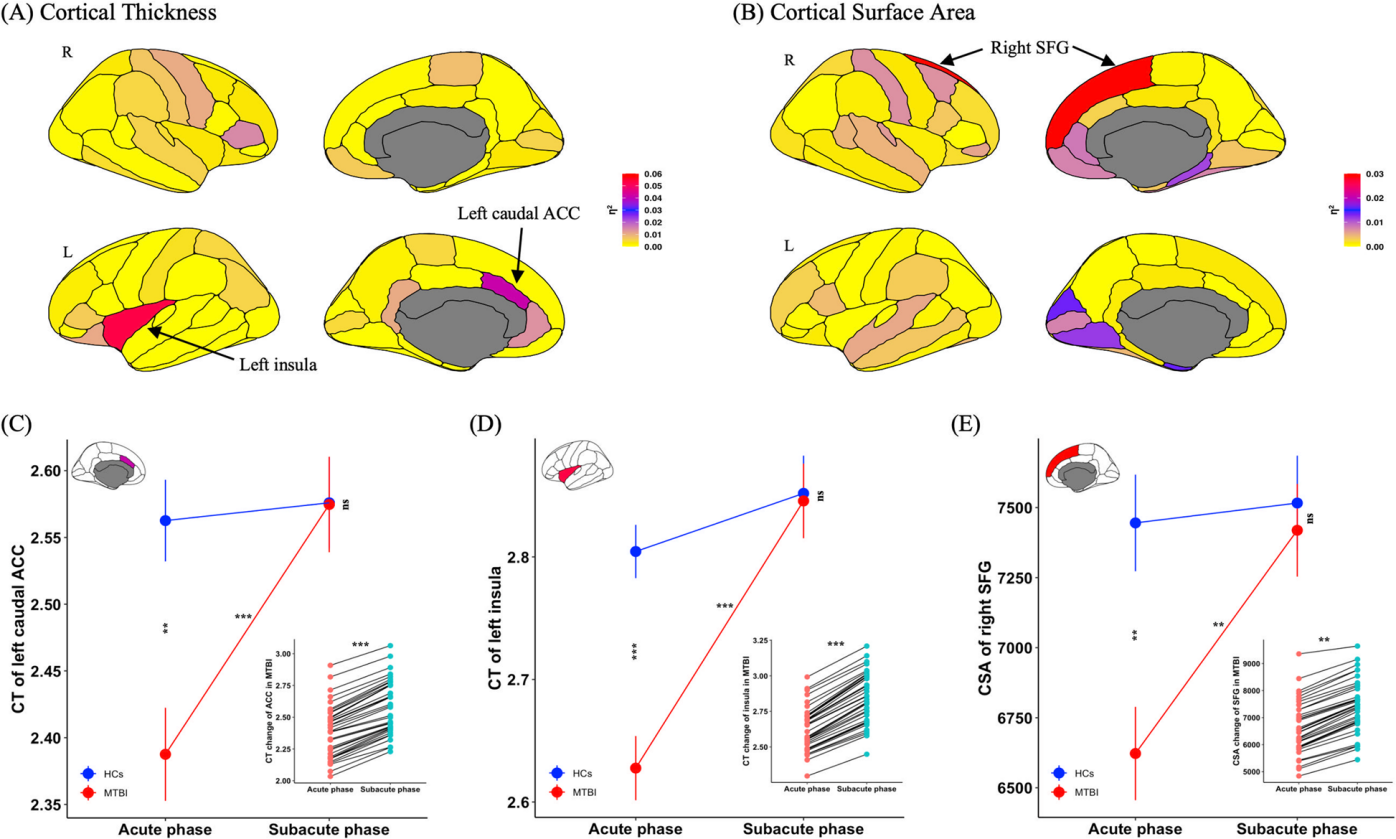
Neuroanatomic map of effect size of groups-by-timepoints interaction effect in (**A**) CT, and (**B**) CSA of 68 bilateral cortical regions. The CT of left caudal ACC and left insula and the CSA of SFG showed high η^2^ for significant interaction effect (*P* < 0.05). Cortical regions are color-coded in correspondence to the effect size (η^2^ value) of CT or CSA. Red-shaded cortical regions indicate high η^2^ for significant interaction effect (*P* < 0.05), while yellow-shaded cortical regions indicate low η^2^ without significant interaction effect. Simple effect analysis of hub neuroanatomical regions included (**C**) CT of left caudal ACC, (**D**) CT of left insula, and (**E**) CSA of right SFG after significant interaction effect was observed. The subpanels in (**C**), (**D**) and (**E**) show neuroanatomical change for each patient from acute to subacute phase, which indicates significant neuroanatomical restoration in ACC, insula and SFG respectively from acute to subacute phase in patients. MTBI, mild traumatic brain injury; PTH, posttraumatic headache; HCs, healthy controls; CT, cortical thickness; CSA, cortical surface area; ACC, anterior cingulate cortex; SFG, superior frontal gyrus; L, left hemisphere; R, right hemisphere; η^2^, Eta Squared Effect Size. ^**^, *P* < 0.01; ^***^, *P* < 0.001; ns, not significant. Data are presented as mean ± SEM

Two-way ANOVA analyses were conducted to investigate CT and CSA development in patients related to HCs from the acute to subacute phase. The effect size in CT of the left caudal ACC and left insula was large (η^2^ $=$ 0.04, 0.05 respectively) with a significant interaction effect (*P* $<$ 0.05, Fig. 3A). For CSA, only the right SFG showed a large effect size (η^2^ $=$ 0.03) with a significant interaction effect (*P* $<$ 0.05, Fig. 3B). There was no significant interaction effect (η^2^ ≈ 0) with CT (Table S1) and CSA (Table S2) in other neuroanatomical regions. Significant main effects of groups (Figure S1) and time points (Figure S2) were observed for CT and CSA in various neuroanatomical regions.

### Neuroanatomical restoration in MTBI with PTH

After a significant interaction effect was observed in three key regions, a simple effect analysis was conducted to examine neuroanatomical restoration in these regions. The simple effect analyses revealed that patients showed lower CT of left caudal ACC compared with HCs during the acute phase (*P* $<$ 0.01), but there was no significant difference between groups during the subacute phase (*P* $>$ 0.05), and patients exhibited CT restoration of left caudal ACC from acute to subacute phase (*P* $<$ 0.001, Fig. 3C). Similarly, patients exhibited lower CT of the left insula compared with HCs during the acute phase (*P* $<$ 0.001), but there was no significant difference between groups during the subacute phase (*P* $>$ 0.05), and patients exhibited CT restoration of the left insula from the acute to subacute phases (*P* $<$ 0.001, Fig. 3D). The same restoration pattern was observed in CSA of right SFG in patients (Fig. 3E).

### Mediation effects of neuroanatomical restoration of salience network in MTBI with PTH

**Fig. 4:**
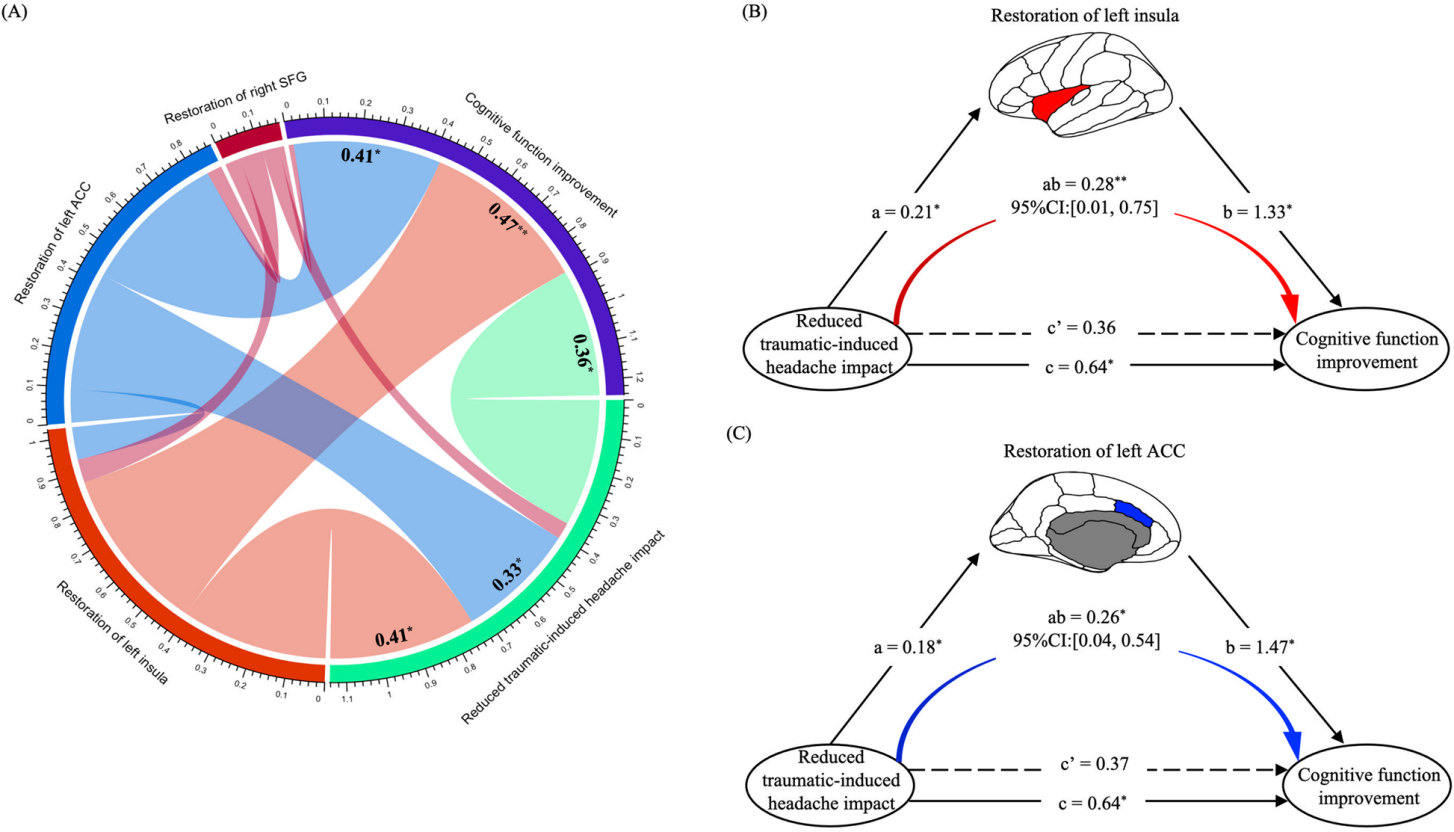
Relationships between the neuroanatomical restoration of hub nodes in salience network, reduced traumatic-induced headache impact and cognitive function improvement in patients. **A** Cognitive function improvement was significantly correlated with restoration of left insula, left ACC, and reduced headache impact; the reduced headache impact was significantly correlated with restoration of left insula, and left ACC. Black numbers represent positive effects. Both the restoration of **B** left insula and **C** left ACC mediated the relationship between reduced headache impact and cognitive function improvement, respectively. MTBI, mild traumatic brain injury; PTH, posttraumatic headache; ACC, anterior cingulate cortex; SFG, superior frontal gyrus; 95% CI, 95% confidence interval; ^*^, *P* < 0.05;.^**^, *P* < 0.01

Correlation analyses showed that cognitive function improvement was positively correlated with both neuroanatomical restoration in the left ACC (r $=$ 0.41, *P* $<$ 0.05) and left insula (*r* $=$ 0.47, *P* $<$ 0.01) and reduced trauma-induced headache (*r* $=$ 0.36, *P* $<$ 0.05) in patients, after controlling for age, sex, and education level. Similarly, trauma-induced headache impact was positively correlated with the neuroanatomical restoration of left ACC (r $=$ 0.33, *P* $<$ 0.05) and left insula (*r* $=$ 0.41, *P* $<$ 0.05) in patients after controlling for age, sex, and education level. However, no significant correlations were found between neuroanatomical restoration in the right SFG and reduced trauma-induced headache impact or cognitive function improvement in patients (all *P* $>$ 0.05, Fig. 4A).

Further mediation analyses found that the effect of reduced trauma-induced headache impact on cognitive function improvement was mediated by neuroanatomical restoration in the left insula (direct effect (c’) $=$ 0.36, *P* $>$ 0.05; indirect effect (ab) $=$ 0.28, 95% CI: [0.01, 0.75], *P* $<$ 0.01, Fig. 4B). Similarly, neuroanatomical restoration in the left ACC had a significant mediating effect on the relationship between reduced trauma-induced headache impact and cognitive function improvement in patients (direct effect (c’) $=$ 0.37, *P* $>$ 0.05; indirect effect (ab) $=$ 0.26, 95% CI: [0.04, 0.54], *P* $<$ 0.05, Fig. 4C).

## Discussion

This study investigated the neuroanatomical restoration in MTBI with PTH from the acute to subacute phases and its relationships with symptomatic improvement. MTBI patients with PTH showed reduced trauma-induced headache impact and improved cognitive function from the acute to subacute phase. Moreover, patients exhibited CT restoration in the left caudal ACC and left insula and CSA restoration in the right SFG from the acute to subacute phases. In addition, CT restoration in the left ACC and insula significantly mediated the relationship between reduced trauma-induced headache impact and cognitive function improvement. These findings suggested that neuroanatomical restoration of key regions in the salience network linked reduced headache to cognitive function improvement in MTBI from the acute to subacute phases, substantiating that the salience network plays an important role in modulating headache impact and cognitive function in MTBI with PTH.

Although trauma-induced headache was more serious in patients during the acute phase than in HCs, no significant difference was found between MTBI and HCs during the subacute phase. This finding implied that patients experienced reduced headaches from the acute to subacute phases. This finding was consistent with previous research, which revealed that cortical glia is persistently activated in the short term after TBI [42], which leads to a proinflammatory cytokine response, followed by proinflammatory cytokines that promote inflammation as well as neuropathic pain, increasing neuro sensitivity and reducing the effects of pain on patients [43, 44]. Similarly, we found significant cognitive function improvement in MTBI patients from the acute to subacute phases. An increasing body of evidence suggests that recovery from the physiological effects of MTBI can be observed at two weeks post-injury [45, 46], and it has also been suggested that cognitive deficits following MTBI were most severe during the short acute period post-injury, but were followed by a rapid recovery to normal levels in one to three months [47, 48].

In addition, compared to HCs, patients showed significantly lower CT of the left caudal ACC and left insula during the acute phase. Overwhelming evidence substantiates that MTBI patients showed altered CT in patients following trauma [49, 50], associated with the severity of the injury, time since injury, local hemorrhage, and regional edema [25, 49]. Given the tight correlation between CT and neuronal density, a decrease in CT is often associated with neuronal damage or reduction [35]. Additionally, animal studies have substantiated that during the acute phase of MTBI, although mild cell death is observed, it triggers extensive dendritic degeneration and reduced synapses in the cortex, leading to atrophic changes in cortical areas [51]. Hence, we hypothesized that CT reduction in the left ACC and left insula might result from reduced neuronal cell density in these regions after MTBI. Similarly, we found that compared to HCs, patients exhibited lower CSA of SFG during the acute phase. However, a previous study found significantly lower GMV of right SFG in individuals after MTBI. This finding suggested that as an integral part of GMV, the reduction of CSA might likely be closely associated with a decrease in total GMV in this region [52]. Furthermore, in the present study, neuroanatomical restoration in the left ACC and insula, and right SFG was observed in MTBI from the acute to subacute phases, consistent with the literature [50, 53] since damage to the adult brain triggered an extensive process of repair and reorganization of surviving neural circuits including widespread brain regions [54]. Taken together, these findings suggest that structural alterations of brain regions during the acute phase of MTBI can be restored to normal levels in this patient population.

Our study found that CT restoration of the left ACC and left insula significantly mediated the relationship between reduced headache impact and cognitive function improvement. As key regions of salience network, ACC and insula are responsible for detecting sensory stimuli and coordinating the switch between the default mode network and executive network [55]. Furthermore, it has been found that pain information input is transmitted via the posterior insula to the anterior insula [56], thus participating in the encoding of pain intensity, while ACC is interconnected with other emotion-related limbic structures that together mediate the motivational and affective dimensions of pain experience [57]. Accordingly, the insula and ACC are important parts of the pain modulation pathway [58]. Previous research has found that lower GMV in the ACC and insula are linked to increased pain sensitivity in individuals [59, 60]. Moreover, altered structural and functional connectivity of key regions ACC and insula in salience network correlated with pain severity in patients with classic trigeminal neuralgia [61]. It has been suggested that reduced CT may imply a reduction in neurons, loss of myelin or other important cells [62], and morphometry abnormalities are potential predictors of subsequent cognitive deficits [63]. Notably, the insula has been established as a key node involved in subjective sensations, including pain, and an important key region for monitoring, attention, and cognitive control, with extensive structural and functional connections with the frontal cortex, ACC, and amygdala [64]. Moreover, the ACC has been regarded as an important region for cognitive function [65]. Previous studies has found that verbal working memory and pain scores were significant correlated with local brain morphology in ACC in fibromyalgia patients [66], and decreased grey matter volume in the pain system (including ACC) are associated with fibromyalgia [67]. In contrast with research showing a significant correlation between diffuse atrophy in several brain regions (including the frontal lobe, ACC, and insula) and cognitive function in individuals following TBI [15, 16], a study found increased GMV in the ventral medial prefrontal cortex, and concomitant improvement in cognitive function as MTBI patients recovered [18]. These findings suggest that reduced pain experienced by patients may be attributed to a decrease in pain sensitivity, which is closely related to the gradual functional recovery of key regions within the salience network, while the neuroanatomical restoration of the left insula and left ACC further reveals the neural remodeling process from acute to subacute phase, which provides an important physiological basis for cognitive improvement in MTBI.

The limitations of the present study should be acknowledged. First, this study only focused on neuroanatomical restoration from the acute to subacute phase in MTBI patients. Future longitudinal studies with more time points post-injury should be conducted to explore the restoration trajectory of the relationships between neuroanatomical features and cognitive function following MTBI. Besides, this study did not consider potential confounders such as previous head trauma and type of brain injury, which should be considered in future research. Besides, the present study results are limited only to structural MRI data. More studies are warranted to assess the validity of the functional restoration of the salience network with functional MRI data collected during resting state or cognitive task (e.g., working memory).

## Conclusions

In summary, using a surface-based morphology approach and mixed ANOVA models, this study indicated that neuroanatomical restoration of key regions in salience network links reduced headache impact to improved cognitive function in MTBI with PTH. Our findings corroborate the vital role of salience network in reduced headache impact and cognitive function improvement in MTBI with PTH during recovery, providing preliminary evidence of the neuroanatomical mechanisms underlying cognitive improvement and headache impact reduction and providing an alternative clinical target in this patient population.

## Supplementary Information

**Additional file 1: Figure S1.** Neuroanatomic map of effect size of (A) groups main effect and (B) timepoints main effect of cortical thickness (CT)in MTBI patients with PTH and HCs. η^2^, Eta Squared Effect Size for ANOVA. **Figure S2.** Neuroanatomic map of effect size of (A) groups main effect and (B) timepoints main effect of cortical surface area (CSA) in MTBI patients with PTH and HCs. η^2^, Eta Squared Effect Size for ANOVA. **Table S1.** Statistics of groups effect, timepoints effect and groups-by-timepoints interaction effect of cortical thickness between MTBI patients with PTH and HCs. **Table S2.** Statistics of groups effect, timepoints effect and groups-by-timepoints interaction effect of cortical surface area between MTBI patients with PTH and HCs. 

## Data Availability

The data that support the findings of this study are available on request from the corresponding authors.

## Abbreviations

TBI: Traumatic brain injury
MTBI: Mild traumatic brain injury
PTH: Posttraumatic headache
CT: Cortical thickness
ACC: Anterior cingulate cortex
GMV: Grey matter volume
CSA: Cortical surface area
ANOVA: Analysis of variance
HCs: Healthy controls
MRI: Magnetic resonance imaging
DSC: Digit Symbol Coding
HIT: Headache Impact Test
η^2^: Eta squared
CI: Confidence interval
SFG: Superior frontal gyrus
