# Supplementary material for: Neuroanatomical restoration of salience network links reduced headache impact to cognitive function improvement in mild traumatic brain injury with posttraumatic headache
Source: J Headache Pain. 2023 Apr 21;24(1):43. doi: 10.1186/s10194-023-01579-0 (PMC10120179; doi:10.1186/s10194-023-01579-0)
Supplement: Supplementary file 1 — Additional file 1: Figure S1. Neuroanatomic map of effect size of (A) groups main effect and (B) timepoints main effect of cortical thickness (CT)in MTBI patients with PTH and HCs. η2, Eta Squared Effect Size for ANOVA. Figure S2. Neuroanatomic map of effect size of (A) groups main effect and (B) timepoints main effect of cortical surface area (CSA) in MTBI patients with PTH and HCs. η2, Eta Squared Effect Size for ANOVA. Table S1. Statistics of groups effect, timepoints effect and groups-by-timepoints interaction effect of cortical thickness between MTBI patients with PTH and HCs. Table S2. Statistics of groups effect, timepoints effect and groups-by-timepoints interaction effect of cortical surface area between MTBI patients with PTH and HCs. [file 10194_2023_1579_MOESM1_ESM.docx]

*Supplementary materials*

***Figure S1.*** Neuroanatomic map of effect size of (A) groups main effect and (B) timepoints main effect

of cortical thickness (CT) in MTBI patients with PTH and HCs. η^2^, Eta Squared Effect Size for ANOVA.





***Figure S2.*** Neuroanatomic map of effect size of (A) groups main effect and (B) timepoints main effect

of cortical surface area (CSA) in MTBI patients with PTH and HCs. η^2^, Eta Squared Effect Size for ANOVA.





| **Table S1**. Statistics of groups effect, timepoints effect and groups-by-timepoints interaction effect of cortical thickness between MTBI patients with PTH and HCs. | | | | | | | | | | | | | | | | | | | | | | |
| --- | --- | --- | --- | --- | --- | --- | --- | --- | --- | --- | --- | --- | --- | --- | --- | --- | --- | --- | --- | --- | --- | --- |
| Brain cortical regions | |  | Groups | | | | |  |  |  |  | Statistics | | | | | | | | | | |
|  |  | MTBI patients with PTH (n=36) | | | |  | HCs (n=34) | | | |  | *Groups effect* | | |  | *Timepoints effect* | | |  | *Groups-by-Timepoints interaction effect* | | |
| *Timepoints* | | *Acute phase* | | *Subacute phase* | |  | *Acute phase* | | *Subacute phase* | |  | *F* | *P* | *η2* |  | *F* | *P* | *η2* |  | *F* | *P* | *η2* |
| *Hemisphere* | *Region* | *Mean* | *SEM* | *Mean* | *SEM* |  | *Mean* | *SEM* | *Mean* | *SEM* |  |  |  |  |  |  |  |  |  |  |  |  |
| Left | Banks of the Superior Temporal Sulcus | 2.18 | 0.03 | 2.10 | 0.02 |  | 2.18 | 0.03 | 2.08 | 0.02 |  | 0.24 | 0.62 | 0.00 |  | 11.24 | 0.00 | 0.08 |  | 0.02 | 0.87 | 0.00 |
| Left | Caudal Anterior Cingulate Cortex | 2.39 | 0.03 | 2.57 | 0.04 |  | 2.56 | 0.03 | 2.58 | 0.03 |  | 6.72 | 0.01 | 0.04 |  | 9.13 | 0.00 | 0.06 |  | 6.53 | 0.01 | 0.04 |
| Left | Caudal Middle Frontal | 2.56 | 0.03 | 2.54 | 0.03 |  | 2.61 | 0.03 | 2.57 | 0.03 |  | 2.91 | 0.09 | 0.02 |  | 1.43 | 0.23 | 0.01 |  | 0.12 | 0.73 | 0.00 |
| Left | Cuneus | 1.95 | 0.02 | 1.95 | 0.02 |  | 1.92 | 0.02 | 1.92 | 0.02 |  | 2.11 | 0.15 | 0.02 |  | 0.03 | 0.86 | 0.00 |  | 0.00 | 0.97 | 0.00 |
| Left | Entorhinal Cortex | 2.47 | 0.07 | 2.65 | 0.07 |  | 2.51 | 0.06 | 2.72 | 0.06 |  | 0.66 | 0.42 | 0.00 |  | 8.74 | 0.00 | 0.06 |  | 0.05 | 0.82 | 0.00 |
| Left | Fusiform Gyrus | 2.39 | 0.02 | 2.26 | 0.02 |  | 2.38 | 0.02 | 2.26 | 0.02 |  | 0.07 | 0.79 | 0.00 |  | 26.19 | 0.00 | 0.16 |  | 0.08 | 0.78 | 0.00 |
| Left | Inferior parietal Lobule | 2.11 | 0.02 | 2.08 | 0.02 |  | 2.08 | 0.03 | 2.08 | 0.02 |  | 0.56 | 0.45 | 0.00 |  | 0.31 | 0.58 | 0.00 |  | 0.74 | 0.39 | 0.01 |
| Left | Inferior Temporal Gyrus | 2.15 | 0.02 | 2.20 | 0.02 |  | 2.15 | 0.02 | 2.20 | 0.02 |  | 0.00 | 1.00 | 0.00 |  | 7.23 | 0.01 | 0.05 |  | 0.00 | 0.96 | 0.00 |
| Left | Isthmus of the Cingulate | 2.18 | 0.02 | 2.22 | 0.03 |  | 2.20 | 0.02 | 2.18 | 0.03 |  | 0.13 | 0.72 | 0.00 |  | 0.30 | 0.59 | 0.00 |  | 1.45 | 0.23 | 0.01 |
| Left | Lateral Occipital Gyrus | 1.83 | 0.02 | 1.82 | 0.02 |  | 1.81 | 0.02 | 1.80 | 0.01 |  | 1.83 | 0.18 | 0.01 |  | 0.37 | 0.54 | 0.00 |  | 0.01 | 0.91 | 0.00 |
| Left | Lateral Orbitofrontal Cortex | 2.41 | 0.02 | 2.51 | 0.02 |  | 2.45 | 0.02 | 2.50 | 0.02 |  | 0.63 | 0.43 | 0.00 |  | 17.60 | 0.00 | 0.11 |  | 1.66 | 0.20 | 0.01 |
| Left | Lingual Gyrus | 2.09 | 0.02 | 2.03 | 0.02 |  | 2.07 | 0.02 | 2.01 | 0.02 |  | 0.62 | 0.43 | 0.00 |  | 9.22 | 0.00 | 0.06 |  | 0.00 | 0.96 | 0.00 |
| Left | Medial orbitofrontal Cortex | 2.30 | 0.02 | 2.38 | 0.02 |  | 2.35 | 0.02 | 2.42 | 0.02 |  | 4.43 | 0.04 | 0.03 |  | 12.99 | 0.00 | 0.08 |  | 0.00 | 0.95 | 0.00 |
| Left | Middle Temporal Gyrus | 2.28 | 0.03 | 2.42 | 0.02 |  | 2.32 | 0.02 | 2.46 | 0.03 |  | 2.91 | 0.09 | 0.02 |  | 29.05 | 0.00 | 0.17 |  | 0.00 | 0.96 | 0.00 |
| Left | Parahippocampal Gyrus | 2.40 | 0.03 | 2.37 | 0.04 |  | 2.44 | 0.03 | 2.38 | 0.04 |  | 0.58 | 0.45 | 0.00 |  | 1.37 | 0.24 | 0.01 |  | 0.12 | 0.73 | 0.00 |
| Left | Paracentral Gyrus | 2.53 | 0.03 | 2.51 | 0.02 |  | 2.47 | 0.03 | 2.50 | 0.02 |  | 2.13 | 0.15 | 0.02 |  | 0.00 | 1.00 | 0.00 |  | 0.68 | 0.41 | 0.00 |
| Left | Pars Opercularis | 2.44 | 0.02 | 2.49 | 0.02 |  | 2.48 | 0.02 | 2.52 | 0.03 |  | 1.87 | 0.17 | 0.01 |  | 3.09 | 0.08 | 0.02 |  | 0.05 | 0.82 | 0.00 |
| Left | Pars Orbitalis | 2.41 | 0.03 | 2.51 | 0.03 |  | 2.46 | 0.03 | 2.53 | 0.03 |  | 1.02 | 0.31 | 0.01 |  | 7.48 | 0.01 | 0.05 |  | 0.15 | 0.70 | 0.00 |
| Left | Pars Triangularis | 2.28 | 0.02 | 2.32 | 0.03 |  | 2.33 | 0.02 | 2.32 | 0.03 |  | 1.51 | 0.22 | 0.01 |  | 0.69 | 0.41 | 0.00 |  | 0.88 | 0.35 | 0.01 |
| Left | Pericalcarine Fissure | 1.85 | 0.02 | 1.90 | 0.02 |  | 1.84 | 0.02 | 1.86 | 0.02 |  | 1.12 | 0.29 | 0.01 |  | 2.24 | 0.14 | 0.02 |  | 0.65 | 0.42 | 0.00 |
| Left | Postcentral Gyrus | 1.98 | 0.02 | 1.99 | 0.02 |  | 2.01 | 0.02 | 1.99 | 0.02 |  | 0.60 | 0.44 | 0.00 |  | 0.21 | 0.64 | 0.00 |  | 0.52 | 0.47 | 0.00 |
| Left | Posterior Cingulate | 2.42 | 0.02 | 2.40 | 0.03 |  | 2.43 | 0.02 | 2.43 | 0.02 |  | 0.76 | 0.39 | 0.01 |  | 0.17 | 0.68 | 0.00 |  | 0.02 | 0.89 | 0.00 |
| Left | Precentral Gyrus | 2.45 | 0.03 | 2.44 | 0.02 |  | 2.48 | 0.03 | 2.48 | 0.02 |  | 1.95 | 0.16 | 0.01 |  | 0.07 | 0.79 | 0.00 |  | 0.02 | 0.88 | 0.00 |
| Left | Precuneus | 2.35 | 0.02 | 2.25 | 0.02 |  | 2.33 | 0.02 | 2.25 | 0.02 |  | 0.14 | 0.71 | 0.00 |  | 24.22 | 0.00 | 0.15 |  | 0.29 | 0.59 | 0.00 |
| Left | Rostral Anterior Cingulate | 2.60 | 0.02 | 2.64 | 0.03 |  | 2.60 | 0.03 | 2.72 | 0.03 |  | 2.04 | 0.16 | 0.01 |  | 7.77 | 0.01 | 0.05 |  | 2.01 | 0.16 | 0.01 |
| Left | Rostral Middle Frontal Gyrus | 2.23 | 0.02 | 2.23 | 0.02 |  | 2.27 | 0.02 | 2.24 | 0.02 |  | 1.80 | 0.18 | 0.01 |  | 0.34 | 0.56 | 0.00 |  | 0.32 | 0.57 | 0.00 |
| Left | Superior Frontal Gyrus | 2.75 | 0.02 | 2.79 | 0.03 |  | 2.80 | 0.02 | 2.82 | 0.02 |  | 2.67 | 0.10 | 0.02 |  | 1.31 | 0.25 | 0.01 |  | 0.12 | 0.73 | 0.00 |
| Left | Superior Parietal Lobule | 2.12 | 0.02 | 2.05 | 0.02 |  | 2.10 | 0.02 | 2.06 | 0.02 |  | 0.29 | 0.59 | 0.00 |  | 8.74 | 0.00 | 0.06 |  | 0.72 | 0.40 | 0.00 |
| Left | Superior Temporal Gyrus | 2.36 | 0.02 | 2.48 | 0.03 |  | 2.39 | 0.03 | 2.50 | 0.02 |  | 1.39 | 0.24 | 0.01 |  | 22.83 | 0.00 | 0.14 |  | 0.04 | 0.84 | 0.00 |
| Left | Supramarginal Gyrus | 2.17 | 0.03 | 2.16 | 0.03 |  | 2.20 | 0.02 | 2.19 | 0.03 |  | 1.53 | 0.22 | 0.01 |  | 0.19 | 0.66 | 0.00 |  | 0.00 | 0.96 | 0.00 |
| Left | Frontal Pole | 2.60 | 0.04 | 2.72 | 0.05 |  | 2.61 | 0.04 | 2.76 | 0.04 |  | 0.31 | 0.58 | 0.00 |  | 9.83 | 0.00 | 0.07 |  | 0.08 | 0.78 | 0.00 |
| Left | Temporal Pole | 2.69 | 0.06 | 2.88 | 0.06 |  | 2.64 | 0.06 | 3.00 | 0.05 |  | 0.36 | 0.55 | 0.00 |  | 21.34 | 0.00 | 0.13 |  | 1.86 | 0.17 | 0.01 |
| Left | Transverse Temporal Cortex | 2.32 | 0.03 | 2.36 | 0.02 |  | 2.36 | 0.02 | 2.41 | 0.03 |  | 3.59 | 0.06 | 0.03 |  | 3.16 | 0.08 | 0.02 |  | 0.00 | 0.95 | 0.00 |
| Left | Insula | 2.63 | 0.03 | 2.85 | 0.03 |  | 2.80 | 0.02 | 2.85 | 0.03 |  | 10.99 | 0.00 | 0.06 |  | 24.02 | 0.00 | 0.13 |  | 9.56 | 0.00 | 0.05 |
| Right | Banks of the Superior Temporal Sulcus | 2.10 | 0.02 | 2.18 | 0.03 |  | 2.10 | 0.02 | 2.18 | 0.02 |  | 0.01 | 0.93 | 0.00 |  | 11.34 | 0.00 | 0.08 |  | 0.03 | 0.87 | 0.00 |
| Right | Caudal Anterior Cingulate | 2.49 | 0.04 | 2.43 | 0.03 |  | 2.52 | 0.04 | 2.45 | 0.02 |  | 0.58 | 0.45 | 0.00 |  | 3.34 | 0.07 | 0.02 |  | 0.01 | 0.93 | 0.00 |
| Right | Caudal Middle Frontal | 2.55 | 0.03 | 2.57 | 0.02 |  | 2.57 | 0.03 | 2.62 | 0.02 |  | 1.77 | 0.19 | 0.01 |  | 2.48 | 0.12 | 0.02 |  | 0.40 | 0.53 | 0.00 |
| Right | Cuneus | 1.96 | 0.02 | 1.97 | 0.02 |  | 1.91 | 0.02 | 1.94 | 0.02 |  | 3.63 | 0.06 | 0.03 |  | 1.07 | 0.30 | 0.01 |  | 0.36 | 0.55 | 0.00 |
| Right | Entorhinal Cortex | 2.46 | 0.06 | 2.42 | 0.08 |  | 2.54 | 0.06 | 2.50 | 0.07 |  | 1.33 | 0.25 | 0.01 |  | 0.45 | 0.51 | 0.00 |  | 0.00 | 0.98 | 0.00 |
| Right | Fusiform Gyrus | 2.26 | 0.02 | 2.41 | 0.02 |  | 2.29 | 0.03 | 2.41 | 0.02 |  | 0.26 | 0.61 | 0.00 |  | 32.88 | 0.00 | 0.19 |  | 0.37 | 0.54 | 0.00 |
| Right | Inferior parietal Lobule | 2.10 | 0.02 | 2.11 | 0.03 |  | 2.09 | 0.02 | 2.11 | 0.03 |  | 0.01 | 0.92 | 0.00 |  | 0.24 | 0.63 | 0.00 |  | 0.14 | 0.71 | 0.00 |
| Right | Inferior Temporal Gyrus | 2.17 | 0.02 | 2.13 | 0.02 |  | 2.16 | 0.02 | 2.09 | 0.02 |  | 1.14 | 0.29 | 0.01 |  | 6.43 | 0.01 | 0.04 |  | 0.58 | 0.45 | 0.00 |
| Right | Isthmus of the Cingulate | 2.23 | 0.03 | 2.22 | 0.03 |  | 2.23 | 0.02 | 2.22 | 0.02 |  | 0.01 | 0.91 | 0.00 |  | 0.14 | 0.71 | 0.00 |  | 0.03 | 0.86 | 0.00 |
| Right | Lateral Occipital Gyrus | 1.81 | 0.02 | 1.85 | 0.02 |  | 1.83 | 0.01 | 1.85 | 0.02 |  | 0.43 | 0.52 | 0.00 |  | 3.26 | 0.07 | 0.02 |  | 0.10 | 0.75 | 0.00 |
| Right | Lateral Orbitofrontal Cortex | 2.47 | 0.02 | 2.42 | 0.02 |  | 2.45 | 0.02 | 2.42 | 0.01 |  | 0.86 | 0.36 | 0.01 |  | 5.47 | 0.02 | 0.04 |  | 0.48 | 0.49 | 0.00 |
| Right | Lingual Gyrus | 2.01 | 0.02 | 2.12 | 0.02 |  | 2.03 | 0.02 | 2.11 | 0.02 |  | 0.09 | 0.77 | 0.00 |  | 22.35 | 0.00 | 0.14 |  | 0.45 | 0.50 | 0.00 |
| Right | Medial orbitofrontal Cortex | 2.39 | 0.02 | 2.35 | 0.02 |  | 2.37 | 0.02 | 2.36 | 0.02 |  | 0.11 | 0.74 | 0.00 |  | 1.90 | 0.17 | 0.01 |  | 0.89 | 0.35 | 0.01 |
| Right | Middle Temporal Gyrus | 2.30 | 0.03 | 2.17 | 0.02 |  | 2.30 | 0.04 | 2.14 | 0.02 |  | 0.34 | 0.56 | 0.00 |  | 26.51 | 0.00 | 0.16 |  | 0.14 | 0.71 | 0.00 |
| Right | Parahippocampal Gyrus | 2.32 | 0.04 | 2.41 | 0.04 |  | 2.36 | 0.03 | 2.45 | 0.03 |  | 1.42 | 0.24 | 0.01 |  | 6.97 | 0.01 | 0.05 |  | 0.00 | 0.99 | 0.00 |
| Right | Paracentral Gyrus | 2.52 | 0.02 | 2.53 | 0.02 |  | 2.51 | 0.02 | 2.56 | 0.02 |  | 0.07 | 0.80 | 0.00 |  | 2.47 | 0.12 | 0.02 |  | 1.13 | 0.29 | 0.01 |
| Right | Pars Opercularis | 2.48 | 0.02 | 2.43 | 0.02 |  | 2.51 | 0.02 | 2.48 | 0.02 |  | 2.99 | 0.09 | 0.02 |  | 3.41 | 0.07 | 0.02 |  | 0.14 | 0.71 | 0.00 |
| Right | Pars Orbitalis | 2.45 | 0.03 | 2.40 | 0.02 |  | 2.47 | 0.03 | 2.40 | 0.03 |  | 0.27 | 0.60 | 0.00 |  | 4.95 | 0.03 | 0.04 |  | 0.04 | 0.85 | 0.00 |
| Right | Pars Triangularis | 2.30 | 0.03 | 2.25 | 0.02 |  | 2.28 | 0.02 | 2.29 | 0.02 |  | 0.12 | 0.73 | 0.00 |  | 1.40 | 0.24 | 0.01 |  | 2.03 | 0.16 | 0.01 |
| Right | Pericalcarine Fissure | 1.85 | 0.02 | 1.86 | 0.02 |  | 1.87 | 0.02 | 1.84 | 0.02 |  | 0.00 | 0.98 | 0.00 |  | 0.24 | 0.63 | 0.00 |  | 0.75 | 0.39 | 0.01 |
| Right | Postcentral Gyrus | 2.01 | 0.02 | 1.99 | 0.02 |  | 1.97 | 0.02 | 1.99 | 0.02 |  | 0.95 | 0.33 | 0.01 |  | 0.02 | 0.89 | 0.00 |  | 0.97 | 0.33 | 0.01 |
| Right | Posterior Cingulate | 2.42 | 0.02 | 2.46 | 0.02 |  | 2.44 | 0.02 | 2.45 | 0.02 |  | 0.02 | 0.89 | 0.00 |  | 1.65 | 0.20 | 0.01 |  | 0.30 | 0.59 | 0.00 |
| Right | Precentral Gyrus | 2.45 | 0.03 | 2.45 | 0.02 |  | 2.44 | 0.03 | 2.51 | 0.02 |  | 0.93 | 0.34 | 0.01 |  | 1.97 | 0.16 | 0.01 |  | 1.45 | 0.23 | 0.01 |
| Right | Precuneus | 2.29 | 0.02 | 2.38 | 0.02 |  | 2.30 | 0.02 | 2.38 | 0.02 |  | 0.00 | 0.98 | 0.00 |  | 25.82 | 0.00 | 0.16 |  | 0.00 | 0.95 | 0.00 |
| Right | Rostral Anterior Cingulate | 2.66 | 0.03 | 2.64 | 0.03 |  | 2.68 | 0.03 | 2.64 | 0.03 |  | 0.06 | 0.81 | 0.00 |  | 1.28 | 0.26 | 0.01 |  | 0.12 | 0.72 | 0.00 |
| Right | Rostral Middle Frontal Gyrus | 2.26 | 0.02 | 2.25 | 0.02 |  | 2.25 | 0.02 | 2.27 | 0.02 |  | 0.07 | 0.79 | 0.00 |  | 0.05 | 0.82 | 0.00 |  | 0.44 | 0.51 | 0.00 |
| Right | Superior Frontal Gyrus | 2.80 | 0.03 | 2.76 | 0.03 |  | 2.81 | 0.03 | 2.79 | 0.03 |  | 0.66 | 0.42 | 0.00 |  | 1.21 | 0.27 | 0.01 |  | 0.14 | 0.71 | 0.00 |
| Right | Superior Parietal Lobule | 2.09 | 0.02 | 2.13 | 0.02 |  | 2.07 | 0.02 | 2.13 | 0.02 |  | 0.22 | 0.64 | 0.00 |  | 8.64 | 0.00 | 0.06 |  | 0.41 | 0.52 | 0.00 |
| Right | Superior Temporal Gyrus | 2.44 | 0.02 | 2.31 | 0.02 |  | 2.40 | 0.03 | 2.33 | 0.03 |  | 0.12 | 0.73 | 0.00 |  | 16.31 | 0.00 | 0.11 |  | 0.86 | 0.35 | 0.01 |
| Right | Supramarginal Gyrus | 2.19 | 0.02 | 2.20 | 0.02 |  | 2.19 | 0.02 | 2.24 | 0.02 |  | 0.60 | 0.44 | 0.00 |  | 1.46 | 0.23 | 0.01 |  | 0.70 | 0.40 | 0.01 |
| Right | Frontal Pole | 2.66 | 0.04 | 2.60 | 0.03 |  | 2.60 | 0.04 | 2.59 | 0.04 |  | 0.58 | 0.45 | 0.00 |  | 0.73 | 0.39 | 0.01 |  | 0.40 | 0.53 | 0.00 |
| Right | Temporal Pole | 2.80 | 0.06 | 2.55 | 0.06 |  | 2.86 | 0.06 | 2.66 | 0.06 |  | 2.04 | 0.16 | 0.01 |  | 14.94 | 0.00 | 0.10 |  | 0.26 | 0.61 | 0.00 |
| Right | Transverse Temporal Cortex | 2.32 | 0.03 | 2.36 | 0.03 |  | 2.37 | 0.03 | 2.36 | 0.02 |  | 0.65 | 0.42 | 0.00 |  | 0.15 | 0.70 | 0.00 |  | 0.59 | 0.44 | 0.00 |
| Right | Insula | 2.83 | 0.03 | 2.76 | 0.03 |  | 2.83 | 0.03 | 2.79 | 0.03 |  | 0.23 | 0.63 | 0.00 |  | 3.89 | 0.05 | 0.03 |  | 0.43 | 0.51 | 0.00 |
| *Notes.* MTBI, mild traumatic brain injury; PTH, posttraumatic headache; HCs, healthy controls; SEM, Standard Error of the Mean; η2, Eta Squared Effect Size. | | | | | | | | | | | | | | | | | | | | | | |

| **Table S2**. Statistics of groups effect, timepoints effect and groups-by-timepoints interaction effect of cortical surface area between MTBI patients with PTH and HCs. | | | | | | | | | | | | | | | | | | | | | | |
| --- | --- | --- | --- | --- | --- | --- | --- | --- | --- | --- | --- | --- | --- | --- | --- | --- | --- | --- | --- | --- | --- | --- |
| Brain cortical regions | |  | Groups | | | | |  |  |  |  | Statistics | | | | | | | | | | |
|  |  | MTBI patients with PTH (n=36) | | | |  | HCs (n=34) | | | |  | *Groups effect* | | |  | *Timepoints effect* | | |  | *Groups-by-Timepoints interaction effect* | | |
| *Timepoints* | | *Acute phase* | | *Subacute phase* | |  | *Acute phase* | | *Subacute phase* | |  | *F* | *P* | *η2* |  | *F* | *P* | *η2* |  | *F* | *P* | *η2* |
| *Hemisphere* | *Region* | *Mean* | *SEM* | *Mean* | *SEM* |  | *Mean* | *SEM* | *Mean* | *SEM* |  |  |  |  |  |  |  |  |  |  |  |  |
| Left | Banks of the Superior Temporal Sulcus | 1080 | 32 | 1068 | 33 |  | 1084 | 28 | 1054 | 31 |  | 0.03 | 0.86 | 0.00 |  | 0.43 | 0.51 | 0.00 |  | 0.09 | 0.76 | 0.00 |
| Left | Caudal Anterior Cingulate | 724 | 29 | 636 | 21 |  | 731 | 28 | 645 | 24 |  | 0.10 | 0.75 | 0.00 |  | 11.28 | 0.00 | 0.08 |  | 0.00 | 0.96 | 0.00 |
| Left | Caudal Middle Frontal | 2397 | 71 | 2720 | 89 |  | 2372 | 85 | 2679 | 79 |  | 0.16 | 0.69 | 0.00 |  | 15.11 | 0.00 | 0.10 |  | 0.01 | 0.92 | 0.00 |
| Left | Cuneus | 1564 | 33 | 1369 | 34 |  | 1628 | 42 | 1539 | 33 |  | 10.86 | 0.00 | 0.07 |  | 16.31 | 0.00 | 0.10 |  | 2.23 | 0.14 | 0.01 |
| Left | Entorhinal Cortex | 444 | 21 | 370 | 17 |  | 413 | 20 | 393 | 20 |  | 0.05 | 0.82 | 0.00 |  | 6.01 | 0.02 | 0.04 |  | 1.97 | 0.16 | 0.01 |
| Left | Fusiform Gyrus | 2988 | 70 | 2957 | 77 |  | 3039 | 58 | 3114 | 64 |  | 2.35 | 0.13 | 0.02 |  | 0.09 | 0.77 | 0.00 |  | 0.61 | 0.44 | 0.00 |
| Left | Inferior parietal Lobule | 5524 | 153 | 5074 | 116 |  | 5637 | 134 | 5169 | 112 |  | 0.63 | 0.43 | 0.00 |  | 12.44 | 0.00 | 0.08 |  | 0.00 | 0.95 | 0.00 |
| Left | Inferior Temporal Gyrus | 3326 | 85 | 3417 | 86 |  | 3474 | 77 | 3501 | 84 |  | 1.93 | 0.17 | 0.01 |  | 0.52 | 0.47 | 0.00 |  | 0.14 | 0.71 | 0.00 |
| Left | Isthmus of the Cingulate | 1048 | 23 | 1046 | 24 |  | 1067 | 27 | 1085 | 28 |  | 1.28 | 0.26 | 0.01 |  | 0.09 | 0.77 | 0.00 |  | 0.17 | 0.68 | 0.00 |
| Left | Lateral Occipital Gyrus | 5482 | 120 | 5059 | 105 |  | 5787 | 124 | 5442 | 128 |  | 8.38 | 0.00 | 0.05 |  | 10.46 | 0.00 | 0.07 |  | 0.11 | 0.74 | 0.00 |
| Left | Lateral Orbitofrontal Cortex | 2718 | 57 | 2766 | 59 |  | 2762 | 64 | 2859 | 54 |  | 1.36 | 0.25 | 0.01 |  | 1.50 | 0.22 | 0.01 |  | 0.19 | 0.67 | 0.00 |
| Left | Lingual Gyrus | 2983 | 68 | 2773 | 70 |  | 2879 | 63 | 2845 | 68 |  | 0.06 | 0.81 | 0.00 |  | 3.42 | 0.07 | 0.02 |  | 1.68 | 0.20 | 0.01 |
| Left | Medial orbitofrontal Cortex | 1938 | 46 | 1877 | 38 |  | 1969 | 51 | 1933 | 42 |  | 0.96 | 0.33 | 0.01 |  | 1.19 | 0.28 | 0.01 |  | 0.08 | 0.77 | 0.00 |
| Left | Middle Temporal Gyrus | 3199 | 70 | 3321 | 65 |  | 3381 | 83 | 3397 | 84 |  | 2.92 | 0.09 | 0.02 |  | 0.86 | 0.36 | 0.01 |  | 0.49 | 0.48 | 0.00 |
| Left | Parahippocampal Gyrus | 738 | 31 | 735 | 33 |  | 714 | 30 | 685 | 18 |  | 1.68 | 0.20 | 0.01 |  | 0.31 | 0.58 | 0.00 |  | 0.20 | 0.66 | 0.00 |
| Left | Paracentral Gyrus | 1350 | 23 | 1357 | 22 |  | 1358 | 31 | 1370 | 32 |  | 0.16 | 0.69 | 0.00 |  | 0.13 | 0.72 | 0.00 |  | 0.01 | 0.92 | 0.00 |
| Left | Pars Opercularis | 1642 | 45 | 1721 | 55 |  | 1718 | 50 | 1881 | 66 |  | 4.67 | 0.03 | 0.03 |  | 4.82 | 0.03 | 0.03 |  | 0.60 | 0.44 | 0.00 |
| Left | Pars Orbitalis | 685 | 18 | 736 | 16 |  | 710 | 18 | 751 | 19 |  | 1.29 | 0.26 | 0.01 |  | 6.74 | 0.01 | 0.05 |  | 0.09 | 0.76 | 0.00 |
| Left | Pars Triangularis | 1349 | 36 | 1467 | 48 |  | 1401 | 45 | 1541 | 54 |  | 1.87 | 0.17 | 0.01 |  | 7.75 | 0.01 | 0.05 |  | 0.06 | 0.81 | 0.00 |
| Left | Pericalcarine Fissure | 1378 | 41 | 1195 | 43 |  | 1418 | 40 | 1324 | 40 |  | 4.25 | 0.04 | 0.03 |  | 11.62 | 0.00 | 0.08 |  | 1.18 | 0.28 | 0.01 |
| Left | Postcentral Gyrus | 4422 | 99 | 4717 | 98 |  | 4500 | 118 | 4723 | 110 |  | 0.16 | 0.69 | 0.00 |  | 6.01 | 0.02 | 0.04 |  | 0.12 | 0.73 | 0.00 |
| Left | Posterior Cingulate | 1232 | 31 | 1185 | 29 |  | 1223 | 32 | 1203 | 27 |  | 0.02 | 0.89 | 0.00 |  | 1.31 | 0.25 | 0.01 |  | 0.19 | 0.66 | 0.00 |
| Left | Precentral Gyrus | 5360 | 115 | 5618 | 115 |  | 5348 | 127 | 5556 | 123 |  | 0.10 | 0.76 | 0.00 |  | 3.79 | 0.05 | 0.03 |  | 0.04 | 0.84 | 0.00 |
| Left | Precuneus | 3954 | 84 | 3840 | 86 |  | 4006 | 93 | 3917 | 95 |  | 0.52 | 0.47 | 0.00 |  | 1.29 | 0.26 | 0.01 |  | 0.02 | 0.89 | 0.00 |
| Left | Rostral Anterior Cingulate | 783 | 24 | 826 | 29 |  | 773 | 31 | 811 | 28 |  | 0.18 | 0.67 | 0.00 |  | 2.08 | 0.15 | 0.02 |  | 0.01 | 0.93 | 0.00 |
| Left | Rostral Middle Frontal Gyrus | 6168 | 142 | 6961 | 182 |  | 6374 | 219 | 6923 | 195 |  | 0.20 | 0.65 | 0.00 |  | 13.25 | 0.00 | 0.09 |  | 0.43 | 0.51 | 0.00 |
| Left | Superior Frontal Gyrus | 7503 | 170 | 7847 | 201 |  | 7649 | 187 | 7827 | 202 |  | 0.11 | 0.74 | 0.00 |  | 1.92 | 0.17 | 0.01 |  | 0.19 | 0.66 | 0.00 |
| Left | Superior Parietal Lobule | 5925 | 139 | 5745 | 122 |  | 6050 | 164 | 5919 | 137 |  | 1.13 | 0.29 | 0.01 |  | 1.24 | 0.27 | 0.01 |  | 0.03 | 0.86 | 0.00 |
| Left | Superior Temporal Gyrus | 3954 | 88 | 4228 | 73 |  | 4137 | 84 | 4259 | 99 |  | 1.54 | 0.22 | 0.01 |  | 5.36 | 0.02 | 0.04 |  | 0.77 | 0.38 | 0.01 |
| Left | Supramarginal Gyrus | 4293 | 142 | 4762 | 151 |  | 4557 | 157 | 4831 | 126 |  | 1.33 | 0.25 | 0.01 |  | 6.72 | 0.01 | 0.05 |  | 0.45 | 0.50 | 0.00 |
| Left | Frontal Pole | 281 | 7 | 284 | 7 |  | 297 | 7 | 279 | 7 |  | 0.61 | 0.44 | 0.00 |  | 1.05 | 0.31 | 0.01 |  | 2.09 | 0.15 | 0.01 |
| Left | Temporal Pole | 496 | 15 | 514 | 13 |  | 498 | 12 | 519 | 12 |  | 0.06 | 0.80 | 0.00 |  | 2.24 | 0.14 | 0.02 |  | 0.01 | 0.91 | 0.00 |
| Left | Transverse Temporal Cortex | 434 | 11 | 455 | 12 |  | 429 | 12 | 456 | 13 |  | 0.04 | 0.84 | 0.00 |  | 3.99 | 0.05 | 0.03 |  | 0.05 | 0.82 | 0.00 |
| Left | Insula | 2859 | 77 | 2680 | 55 |  | 2780 | 85 | 2574 | 48 |  | 1.87 | 0.17 | 0.01 |  | 8.02 | 0.01 | 0.05 |  | 0.04 | 0.84 | 0.00 |
| Right | Banks of the Superior Temporal Sulcus | 870 | 23 | 867 | 25 |  | 853 | 26 | 887 | 21 |  | 0.00 | 0.95 | 0.00 |  | 0.41 | 0.52 | 0.00 |  | 0.62 | 0.43 | 0.00 |
| Right | Caudal Anterior Cingulate | 689 | 30 | 778 | 34 |  | 721 | 37 | 768 | 33 |  | 0.11 | 0.74 | 0.00 |  | 4.30 | 0.04 | 0.03 |  | 0.40 | 0.53 | 0.00 |
| Right | Caudal Middle Frontal | 2448 | 79 | 2311 | 76 |  | 2535 | 90 | 2238 | 81 |  | 0.01 | 0.93 | 0.00 |  | 6.93 | 0.01 | 0.05 |  | 0.98 | 0.32 | 0.01 |
| Right | Cuneus | 1457 | 41 | 1552 | 33 |  | 1584 | 46 | 1680 | 33 |  | 10.89 | 0.00 | 0.07 |  | 6.11 | 0.01 | 0.04 |  | 0.00 | 0.99 | 0.00 |
| Right | Entorhinal Cortex | 380 | 17 | 359 | 18 |  | 402 | 19 | 408 | 23 |  | 3.37 | 0.07 | 0.02 |  | 0.15 | 0.70 | 0.00 |  | 0.48 | 0.49 | 0.00 |
| Right | Fusiform Gyrus | 2785 | 64 | 2808 | 56 |  | 2974 | 68 | 2870 | 57 |  | 4.16 | 0.04 | 0.03 |  | 0.41 | 0.53 | 0.00 |  | 1.06 | 0.30 | 0.01 |
| Right | Inferior parietal Lobule | 5701 | 139 | 6091 | 148 |  | 5974 | 165 | 6381 | 137 |  | 3.66 | 0.06 | 0.02 |  | 7.34 | 0.01 | 0.05 |  | 0.00 | 0.96 | 0.00 |
| Right | Inferior Temporal Gyrus | 3183 | 97 | 3002 | 93 |  | 3292 | 67 | 3181 | 62 |  | 3.07 | 0.08 | 0.02 |  | 3.19 | 0.08 | 0.02 |  | 0.18 | 0.67 | 0.00 |
| Right | Isthmus of the Cingulate | 1012 | 25 | 1001 | 23 |  | 1035 | 25 | 1000 | 21 |  | 0.22 | 0.64 | 0.00 |  | 0.91 | 0.34 | 0.01 |  | 0.25 | 0.62 | 0.00 |
| Right | Lateral Occipital Gyrus | 4966 | 131 | 5266 | 104 |  | 5342 | 130 | 5520 | 100 |  | 7.18 | 0.01 | 0.05 |  | 4.19 | 0.04 | 0.03 |  | 0.27 | 0.60 | 0.00 |
| Right | Lateral Orbitofrontal Cortex | 2822 | 56 | 2807 | 66 |  | 2994 | 65 | 2910 | 64 |  | 4.78 | 0.03 | 0.03 |  | 0.59 | 0.44 | 0.00 |  | 0.30 | 0.59 | 0.00 |
| Right | Lingual Gyrus | 2925 | 75 | 3021 | 66 |  | 2985 | 88 | 2978 | 60 |  | 0.01 | 0.91 | 0.00 |  | 0.40 | 0.53 | 0.00 |  | 0.50 | 0.48 | 0.00 |
| Right | Medial orbitofrontal Cortex | 2038 | 36 | 1979 | 44 |  | 1994 | 41 | 2021 | 46 |  | 0.00 | 0.98 | 0.00 |  | 0.17 | 0.68 | 0.00 |  | 1.05 | 0.31 | 0.01 |
| Right | Middle Temporal Gyrus | 3603 | 72 | 3386 | 77 |  | 3592 | 83 | 3443 | 74 |  | 0.09 | 0.77 | 0.00 |  | 5.78 | 0.02 | 0.04 |  | 0.19 | 0.66 | 0.00 |
| Right | Parahippocampal Gyrus | 730 | 21 | 652 | 18 |  | 681 | 19 | 651 | 14 |  | 1.89 | 0.17 | 0.01 |  | 9.06 | 0.00 | 0.06 |  | 1.68 | 0.20 | 0.01 |
| Right | Paracentral Gyrus | 1542 | 29 | 1515 | 24 |  | 1535 | 38 | 1530 | 44 |  | 0.01 | 0.91 | 0.00 |  | 0.23 | 0.63 | 0.00 |  | 0.10 | 0.75 | 0.00 |
| Right | Pars Opercularis | 1519 | 48 | 1454 | 41 |  | 1531 | 42 | 1405 | 42 |  | 0.18 | 0.67 | 0.00 |  | 4.67 | 0.03 | 0.03 |  | 0.49 | 0.49 | 0.00 |
| Right | Pars Orbitalis | 875 | 17 | 826 | 20 |  | 867 | 19 | 851 | 16 |  | 0.24 | 0.63 | 0.00 |  | 3.17 | 0.08 | 0.02 |  | 0.79 | 0.38 | 0.01 |
| Right | Pars Triangularis | 1754 | 51 | 1628 | 42 |  | 1736 | 55 | 1593 | 47 |  | 0.29 | 0.59 | 0.00 |  | 7.57 | 0.01 | 0.05 |  | 0.03 | 0.86 | 0.00 |
| Right | Pericalcarine Fissure | 1356 | 52 | 1473 | 41 |  | 1427 | 49 | 1524 | 33 |  | 1.86 | 0.17 | 0.01 |  | 5.80 | 0.02 | 0.04 |  | 0.05 | 0.82 | 0.00 |
| Right | Postcentral Gyrus | 4644 | 97 | 4284 | 93 |  | 4580 | 104 | 4417 | 103 |  | 0.12 | 0.73 | 0.00 |  | 7.10 | 0.01 | 0.05 |  | 0.99 | 0.32 | 0.01 |
| Right | Posterior Cingulate | 1207 | 27 | 1242 | 30 |  | 1193 | 34 | 1198 | 35 |  | 0.86 | 0.36 | 0.01 |  | 0.40 | 0.53 | 0.00 |  | 0.21 | 0.65 | 0.00 |
| Right | Precentral Gyrus | 5346 | 115 | 5152 | 105 |  | 5451 | 134 | 5152 | 107 |  | 0.21 | 0.65 | 0.00 |  | 4.48 | 0.04 | 0.03 |  | 0.21 | 0.65 | 0.00 |
| Right | Precuneus | 4039 | 89 | 4064 | 93 |  | 4072 | 95 | 4110 | 93 |  | 0.18 | 0.67 | 0.00 |  | 0.11 | 0.74 | 0.00 |  | 0.01 | 0.94 | 0.00 |
| Right | Rostral Anterior Cingulate | 643 | 22 | 639 | 21 |  | 637 | 24 | 585 | 21 |  | 1.80 | 0.18 | 0.01 |  | 1.58 | 0.21 | 0.01 |  | 1.17 | 0.28 | 0.01 |
| Right | Rostral Middle Frontal Gyrus | 6715 | 153 | 6117 | 162 |  | 6503 | 155 | 6084 | 171 |  | 0.59 | 0.44 | 0.00 |  | 10.17 | 0.00 | 0.07 |  | 0.31 | 0.58 | 0.00 |
| Right | Superior Frontal Gyrus | 6622 | 167 | 7419 | 165 |  | 7445 | 172 | 7516 | 169 |  | 7.47 | 0.01 | 0.05 |  | 6.97 | 0.01 | 0.04 |  | 4.65 | 0.03 | 0.03 |
| Right | Superior Parietal Lobule | 5671 | 108 | 5668 | 103 |  | 5846 | 129 | 5989 | 165 |  | 3.78 | 0.05 | 0.03 |  | 0.28 | 0.60 | 0.00 |  | 0.33 | 0.57 | 0.00 |
| Right | Superior Temporal Gyrus | 3995 | 79 | 3701 | 84 |  | 3888 | 92 | 3726 | 64 |  | 0.26 | 0.61 | 0.00 |  | 8.13 | 0.01 | 0.06 |  | 0.68 | 0.41 | 0.00 |
| Right | Supramarginal Gyrus | 4438 | 123 | 3944 | 104 |  | 4382 | 117 | 3986 | 118 |  | 0.00 | 0.95 | 0.00 |  | 14.83 | 0.00 | 0.10 |  | 0.18 | 0.68 | 0.00 |
| Right | Frontal Pole | 327 | 6 | 342 | 7 |  | 337 | 8 | 349 | 7 |  | 1.44 | 0.23 | 0.01 |  | 3.70 | 0.06 | 0.03 |  | 0.08 | 0.78 | 0.00 |
| Right | Temporal Pole | 506 | 12 | 483 | 12 |  | 547 | 12 | 511 | 13 |  | 8.10 | 0.01 | 0.05 |  | 5.80 | 0.02 | 0.04 |  | 0.25 | 0.62 | 0.00 |
| Right | Transverse Temporal Cortex | 367 | 11 | 331 | 8 |  | 346 | 11 | 329 | 8 |  | 1.37 | 0.24 | 0.01 |  | 7.42 | 0.01 | 0.05 |  | 0.90 | 0.34 | 0.01 |
| Right | Insula | 2403 | 55 | 2496 | 63 |  | 2366 | 59 | 2510 | 73 |  | 0.03 | 0.86 | 0.00 |  | 3.55 | 0.06 | 0.03 |  | 0.17 | 0.68 | 0.00 |
| *Notes.* MTBI, mild traumatic brain injury; PTH, posttraumatic headache; HCs, healthy controls; SEM, Standard Error of the Mean; η2, Eta Squared Effect Size. | | | | | | | | | | | | | | | | | | | | | | |
